# Overexpression of Rice Wall-Associated Kinase 25 (OsWAK25) Alters Resistance to Bacterial and Fungal Pathogens

**DOI:** 10.1371/journal.pone.0147310

**Published:** 2016-01-21

**Authors:** Mitch Harkenrider, Rita Sharma, David De Vleesschauwer, Li Tsao, Xuting Zhang, Mawsheng Chern, Patrick Canlas, Shimin Zuo, Pamela C. Ronald

**Affiliations:** 1 Department of Plant Pathology, University of California Davis, Davis, California, United States of America; 2 Joint BioEnergy Institute, Emeryville, California, United States of America; 3 Lab of Phytopathology, Ghent University, Ghent, Belgium; 4 Jiangsu Key Laboratory of Crop Genetics and Physiology and Co-Innovation Center for Modern Production Technology of Grain Crops, Yangzhou University, Yangzhou, China; Fujian Agriculture and Forestry University, CHINA

## Abstract

Wall-associated kinases comprise a sub-family of receptor-like kinases that function in plant growth and stress responses. Previous studies have shown that the rice wall-associated kinase, OsWAK25, interacts with a diverse set of proteins associated with both biotic and abiotic stress responses. Here, we show that wounding and BTH treatments induce *OsWAK25* transcript expression in rice. We generated OsWAK25 overexpression lines and show that these lines exhibit a lesion mimic phenotype and enhanced expression of rice *NH1* (NPR1 homolog 1), *OsPAL2*, *PBZ1* and *PR10*. Furthermore, these lines show resistance to the hemibiotrophic pathogens, *Xanthomonas oryzae* pv. *oryzae* (*Xoo*) and *Magnaporthe oryzae*, yet display increased susceptibility to necrotrophic fungal pathogens, *Rhizoctonia solani* and *Cochliobolus miyabeanus*.

## Introduction

Wall-associated kinases (WAKs) comprise a subfamily of proteins within the receptor-like kinase (RLK) superfamily. Though not all WAK and WAK-like proteins encode transmembrane proteins, WAK-RLKs contain an extracellular domain, a transmembrane domain and an intracellular serine/threonine kinase domain [1]. The extracellular domain contains epidermal growth factor-like repeats thus defining the hallmark of a WAK. The extracellular domain of several WAKs has been shown to form a bond with the cell wall that persists during plasmolysis [2, 3], and the intracellular kinase domain is responsible for activating cytoplasmic signaling cascades [4].

The majority of knowledge about WAKs and their roles in signal transduction and pathogen stress responses arose from studies in the plant model, *Arabidopsis thaliana*. Genome-wide microarray-based transcriptomics in *Arabidopsis* implicate WAK involvement during responses to pathogens and establishing systemic acquired resistance (SAR) [5, 6]. SAR is a salicylic acid-dependent response that confers long-lasting and broad-spectrum resistance to viral, fungal, and bacterial pathogens via increased expression of pathogenesis-related genes (PR genes). Several studies have linked WAK genes with pathogen defense and SAR-related responses. For example, WAK1 expression increases following both *Pseudomonas syringae* infection and exogenous application of salicylic acid (SA) [3]. Additionally, an *Arabidopsis* mutant, *npr1*, displayed reduced WAK1 transcript levels after SA application, further supporting an association between WAK1 and SA-mediated pathways [7]. When a WAK1 antisense construct is expressed in *Arabidopsis*, the mRNA level of pathogenesis-related protein 1 (PR1), a gene activated during SAR, is reduced [7]. When overexpressed, WAK1 confers resistance to *Botrytis cinerea* [8]. While WAK1 is the most characterized wall-associated kinase thus far, other WAK genes in *Arabidopsis* have been characterized in response to pathogens as well, notably WAK2 [4], WAKL10 [9], and WAKL22 [9, 10].

Beyond *Arabidopsis*, WAKs have demonstrated critical roles in pathogen responses in other species including, tomato [11], wheat [12], and the model grass, rice. A genome-wide study in rice identified 125 members of the WAK/WAKL family; 67 of these genes encode WAK-RLKs [1]. However, few of these WAK genes have been examined for roles in defense responses. One exception is OsWAK1, shown to be induced by wounding, SA, methyl-jasmonate, and rice blast (*M*. *oryzae*) but not abscisic acid. Furthermore, overexpression of OsWAK1 conferred resistance to *M*. *oryzae* [13]. In another study, a reporter construct containing the OsWAK11 promoter fused to GUS is activated in response to wounding [14].

OsWAK25 (*LOC_Os03g12470*) is one of the 67 WAK-RLKs identified in rice and encodes a 76 kDa protein with one EGF-like repeat in the extracellular domain. The structure of OsWAK25 has two-introns and three-exons, similar to *Arabidopsis* WAK and WAK-like genes [1, 15] and clusters more closely with homologs from *Arabidopsis* and poplar than with any other rice WAK [16]. In a microarray-based transcriptome study, OsWAK25 mRNA was upregulated during the early stages of *M*. *oryzae* infection [17]. Additionally, Jo et al report the localization of OsWAK25 at the plasmodesmata 36 hours following agrobacterium-mediated transient expression in tobacco leaves [2]. The localization signal persists during plasmolysis indicating a covalent connection between OsWAK25 and the cell wall [2, 3]. Two protein-protein interaction studies demonstrated that the OsWAK25 kinase domain associates with a diverse set of proteins that govern responses to abiotic and biotic stress (S1 Fig)[18, 19]. Furthermore, overexpression of OsWAK25 in rice plants containing Xa21 conferred enhanced resistance to *Xoo* compared with plants containing Xa21 alone [19]. These aforementioned studies introduced the importance of OsWAK25 in rice innate immunity. In this study, we aim to determine if OsWAK25 alone has a role in biotic stress responses. Toward this aim, we generated Kitaake rice plants overexpressing OsWAK25, assessed resistance to hemibiotrophic and necrotrophic pathogens and assayed expression of defense-related genes.

## Results

### OsWAK25 has protein domains hallmark of a WAK-RLK

**Fig 1:**
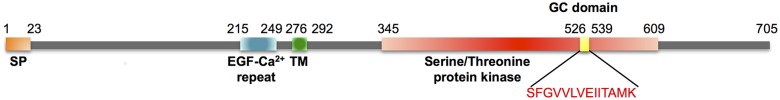
OsWAK25 has the domains characteristic of a WAK-RLK. Protein motifs and domains of OsWAK25 were identified using the Simple Modular Architecture Research Tool (SMART) database (http://smart.embl-heidelberg.de/) and NCBI-CDD (http://www.ncbi.nlm.nih.gov/Structure/cdd/wrpsb.cgi). The GC domain was identified manually. SP, signal peptide (orange); EGF-Ca^2+^ repeat (blue) TM, transmembrane region (green); serine-threonine kinase domain (red); GC, guanylyl cyclase domain (yellow).

As reported previously [1], the extracellular domain of OsWAK25 contains an epidermal growth factor-like repeat (EGF-Ca^2+^), the hallmark of the wall-associated kinase family (Fig 1). We add that the intracellular portion of OsWAK25 has a predicted twin domain containing a guanylyl cyclase [20] sequence nested within a serine/threonine kinase domain. Detailed amino acid sequence and protein information is found in S2 Fig.

### OsWAK25 transcript accumulates after BTH treatment and wounding

**Fig 2:**
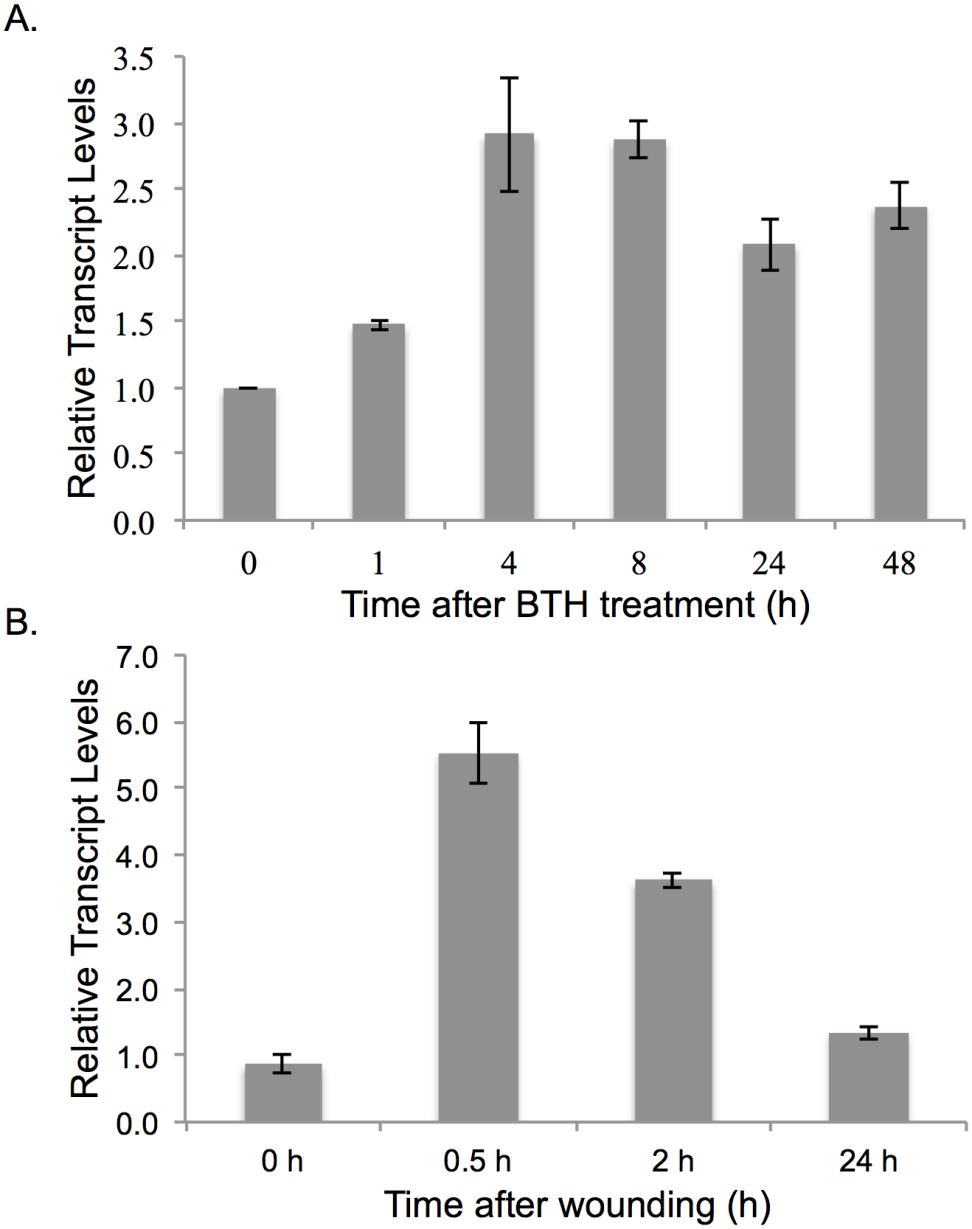
OsWAK25 transcript is induced following BTH treatment and wounding. (A) OsWAK25 transcript in wild type, Kitaake, plants accumulates in response to a foliar spray containing BTH. This response persists up to 48 h post application. (B) Transcript of OsWAK25 increases in response to wounding however the response returns to wild type levels after 24 h. Columns represent the average fold-change of three biological replicates as compared to time = 0 h. C_T_ values were first normalized to actin as an internal control followed by a normalization to the 0 h time point. Each assay was performed twice with similar results.

Salicylic acid (SA) signaling is mimicked by exogenous application of the chemical analogue, benzothiadiazole (BTH). To investigate whether OsWAK25 is involved in SA-mediated responses and transcriptionally influenced by BTH, we applied a foliar spray treatment to mature, fully expanded leaves of the rice cultivar, Kitaake. Fig 2A shows the upregulation of OsWAK25 with the highest transcript levels occurring 4 hours after treatment with the elevated transcript levels persisting up to 48 hours.

To check if OsWAK25 transcript also accumulates in response to wounding, we repeatedly punctured a 3–4 cm leaf segment with a pin needle. We then collected the segments at 30 minutes, 2 hours, and 24 hours following treatment and measured transcript using qPCR (Fig 2B). OsWAK25 is rapidly upregulated in response to wounding, having the highest accumulation only 30 minutes following treatment. Normal transcript levels returned after 24 hours.

### Overexpression of OsWAK25 confers resistance to *Xanthomonas oryzae* pv. *oryzae* and *Magnaporthe oryzae*

To generate OsWAK25 overexpression plants, we transformed the japonica cultivar, Kitaake, with a construct containing *OsWAK25* full-length cDNA driven by the maize ubiquitin promoter (S3 Fig) [19]. Using an established method [21, 22], we obtained 4 independently transformed lines (2, 3, 5 and 10) containing the transgenic construct and one line (4) that contains hygromycin, the selectable marker gene, but not the full overexpression construct.

**Fig 3:**
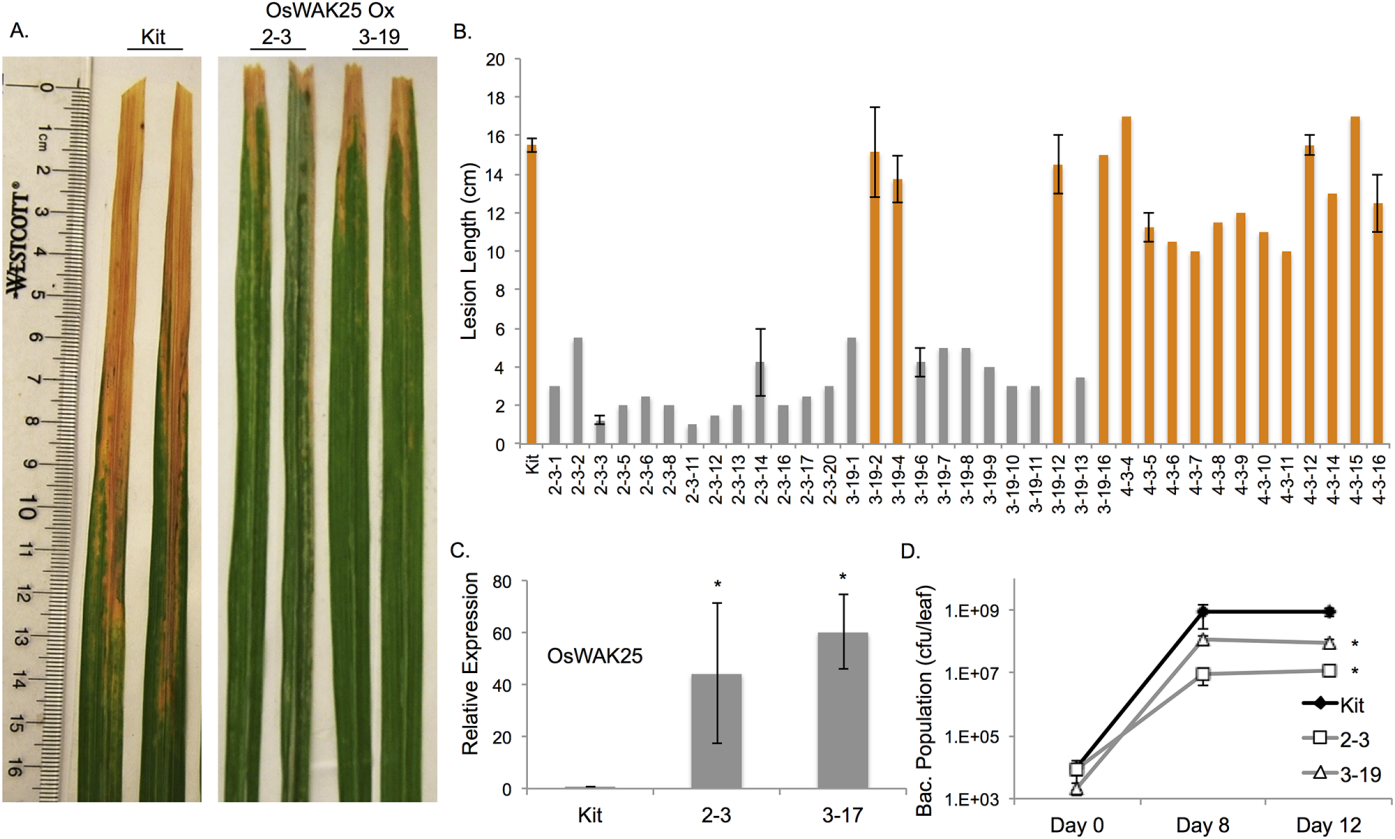
OsWAK25 overexpression confers resistance to *Xanthamonas oryae* pv. *oryzae*. (A) Photos were taken 14 days after scissors inoculation with *Xoo* strain PXO99. (B) Lesion lengths were measured 14 dpi on progeny from overexpression plants 2–3, 3–19, and 4–3. Plants were tested for presence of the overexpression construct (grey) or not (orange) using PCR. Line 4 is a null transformant, not containing the full transgenic construct. (C) qPCR results indicates a large accumulation of *OsWAK25* transcript in progeny from plants 2–3 and 3–17. Each column represents the average and standard error of three plants with three technical replicates. Expression was normalized first to actin, then to Kitaake, giving fold-change relative to the control. (D) Each time point represents the average and standard error of three leaves from sibling progeny of the plant indicated, each with three technical replicates. Lines 3–17 and 3–19 are T_1_ sibling plants. Asterisks represent a significant difference from kitaake plants based on Student’s t-test, p < 0.001. Each experiment was performed at least three times with significant results. Data from only one experimental replicate is shown.

To test whether overexpression of OsWAK25 in a Kitaake background leads to enhanced resistance to bacterial blight disease caused by *Xanthomonas oryzae* pv. *oryzae* (*Xoo*), we challenged six-week old T_2_ progeny from lines 2, 3, and 4 with *Xoo* strain PXO99 using scissors inoculation. Plants were examined for co-segregation of phenotype with genotype by measuring lesion development in mature plants and assessing the presence of the transgene by PCR analysis. OsWAK25 overexpression lines 2–3 and 3–19 exhibited very short lesions, ranging from 1–3 cm, compared to both the Kitaake wild type control and plants lacking the transgene having lesions about 15 cm in length (Fig 3A and 3B). For the data in Fig 3, line 2–3 is homozygous for the overexpression construct, line 3–19 is segregating and line 4 is PCR-negative for the overexpression construct. Based on these results, line 4 was not used for further experiments.

Total RNA from the progeny of T_1_ plants from lines 2 and 3 was collected and tested using qPCR. These overexpression lines express an increased amount of OsWAK25 transcript approximately 40 to 60-fold higher than wild type expression (Fig 3C).

To quantify the enhanced resistance phenotype, we measured bacterial growth within the leaf over 12 days. Lines 2–3 and 3–19 overexpressing OsWAK25 have significantly fewer colony forming units per leaf, 1 × 10^7^ and 9 × 10^7^ respectively, compared to 9 × 10^8^ CFU for the Kitaake control (Fig 3D). These experiments indicate that overexpression of OsWAK25 confers resistance to *Xoo*. To test whether reduced expression of this gene has any effect, we generated transgenic plants containing an OsWAK25 RNAi construct (lines RNAi 2, RNAi 5 and RNAi 6). We tested each line for gene expression and resistance to *Xoo* using both bacterial growth and lesion length assays with three different *Xoo* strains, PXO99, NXO256 and MXO90 (S4 Fig). No statistically significant difference from the Kitaake control was measured in any of these experiments.

**Fig 4:**
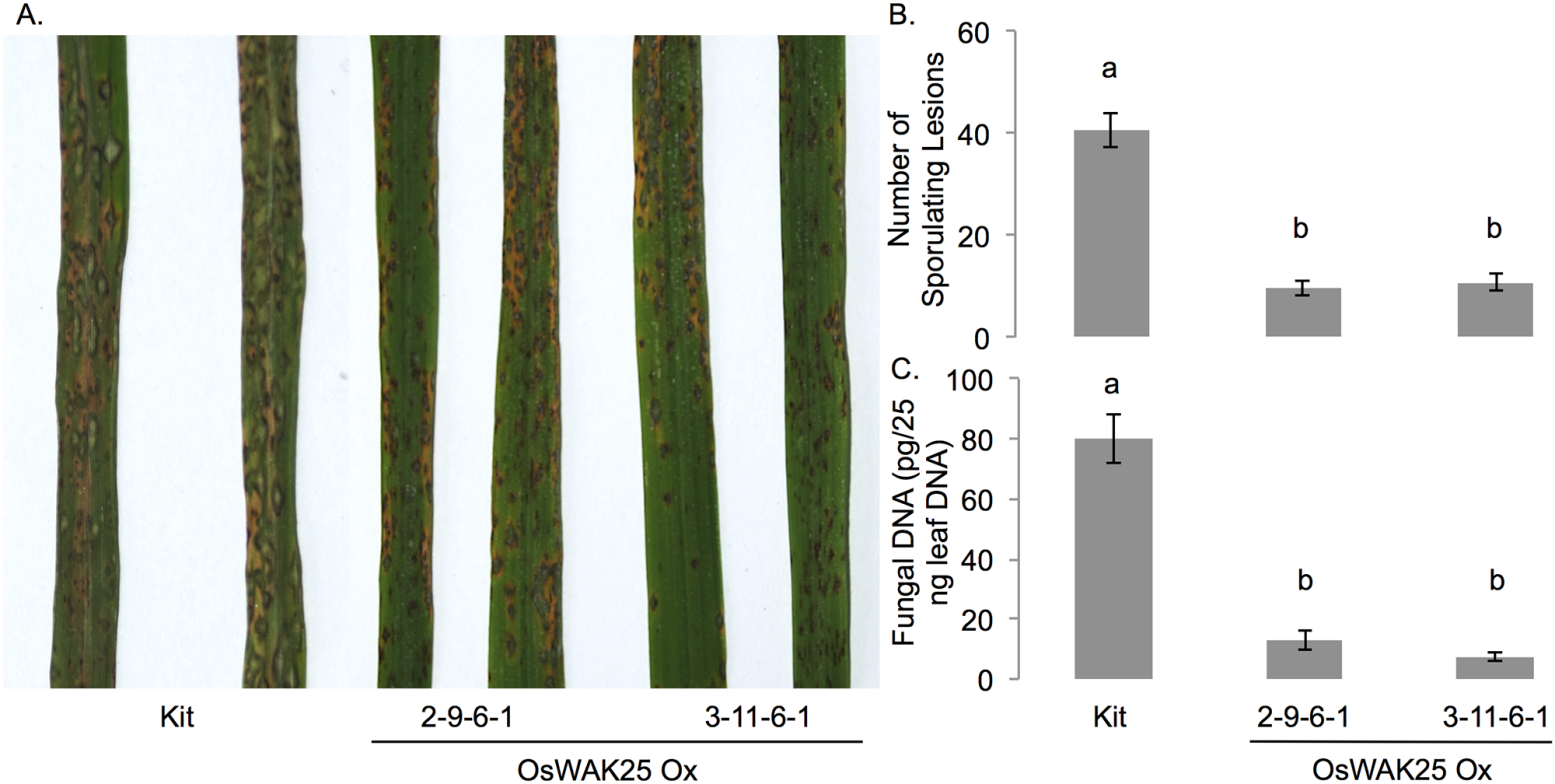
OsWAK25 overexpression confers resistance to *Magnaporthe oryzae*. Four-week old rice plants were inoculated with the highly virulent strain, VT7. (A & B) Photos were taken and sporulating lesions were counted 6 dpi. (C) Fungal DNA was estimated using a qPCR-based method with primers targeting the 28S ribosomal gene. Different letters represent statistical significance from an ANOVA followed by a Tukey-Kramer significance test. These experiments were conducted twice with data from one representative experiment shown.

To test if overexpression of OsWAK25 conferred resistance to other hemibiotrophic pathogens, we challenged overexpression lines 2-9-6-1 and 3-11-6-1 with *M*. *oryzae*, the causal agent of rice blast. An initial experiment was conducted using VT5M1, a strain moderately virulent on rice cultivar Nipponbare, yet no statistically significant difference in the number of lesions between OsWAK25 overexpression lines and Kitaake was measured (data not shown). Subsequent experiments were conducted using VT7, a strain highly virulent on Nipponbare [23]. With this strain, we observed enhanced resistance in overexpression lines 2-9-6-1 and 3-11-6-1 compared with Kitaake (Fig 4). Six days following inoculation, the number of sporulating lesions, identified by a characteristic gray center, was counted. Overexpression lines exhibited approximately 10 lesions per leaf compared to an average of 40 lesions on each Kitaake leaf. To further quantify the infection, we estimated the amount of fungal DNA present in a leaf sample using a qPCR-based strategy [24]. These data indicate an 8-fold reduction in the amount of fungal 28S rDNA when OsWAK25 is overexpressed. In sum, these data indicate that OsWAK25 overexpression provides resistance to hemibiotrophic pathogens.

### OsWAK25 overexpression plants display a lesion mimic phenotype and enhanced expression of NH1 and key defense genes

**Fig 5:**
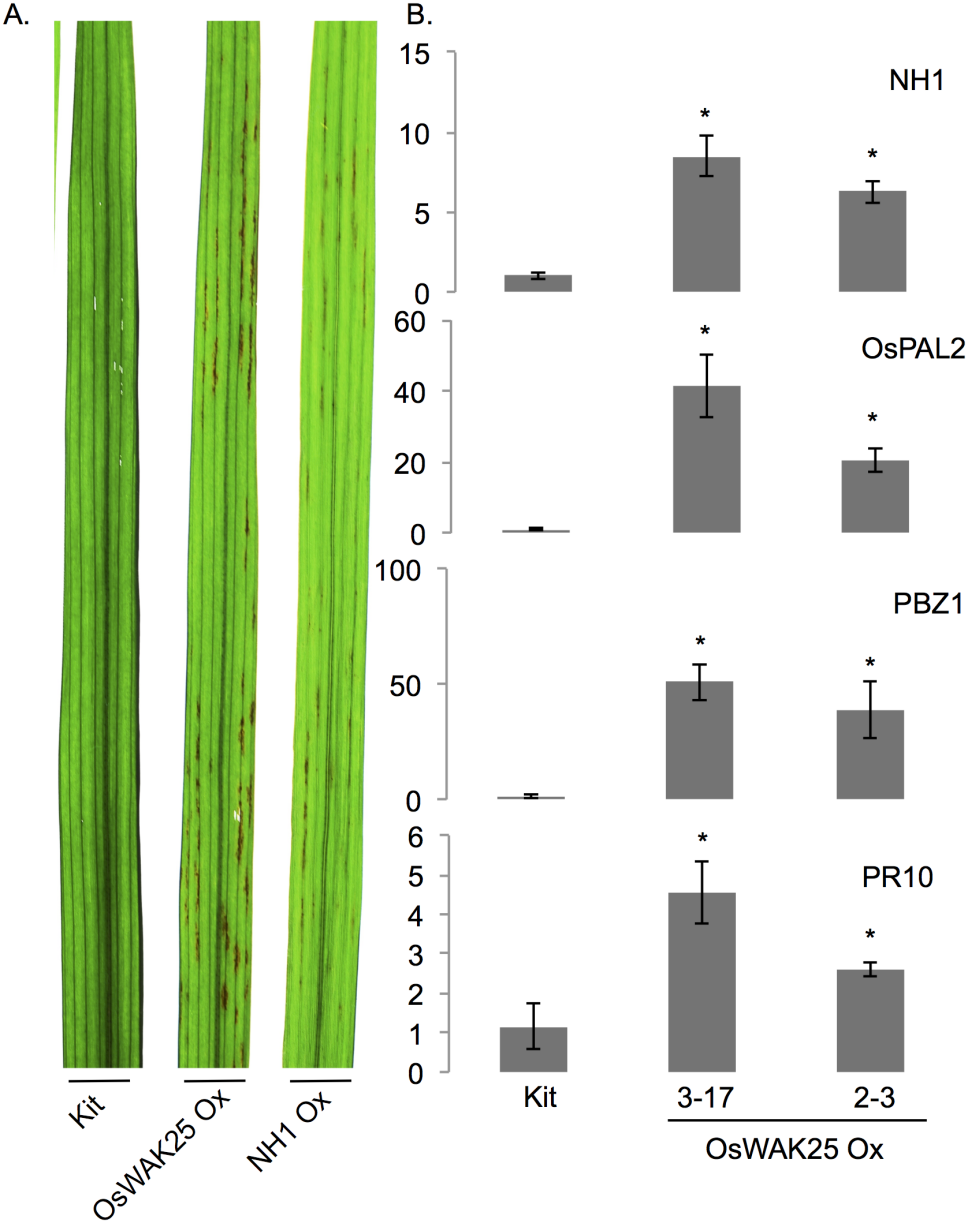
OsWAK25 overexpression plants exhibit mimic lesions and correlate with an upregulation of defense genes. Plants were grown under greenhouse conditions. (A) Leaves collected at six-weeks. OsWAK25 overexpression and NH1 overexpression exhibit similar mimic lesions. Experiment was conducted once with 10 plants for each line. (B) Leaf tissue for RNA extraction was collected from 4-week old progeny from plant lines 3–17 and 2–3 along with Kitaake controls. Y-axes on the graphs indicate fold-change differences from Kitaake. C_T_ values were first normalized to ubiquitin as an internal control followed by normalization to Kitaake. Each bar represents the average and standard error of 3 sibling plants. Asterisks represent statistical significance with Student’s t-test, p < 0.0001. The qPCR assays were conducted twice with similar results.

A constitutively expressed immune response in plants can result in the presence of small necrotic spots on leaves. We observed such spots consistently on fully expanded leaves of mature OsWAK25 overexpression plants, whereas no lesion mimic phenotype was observed on Kitaake plants grown under the same conditions. A similar lesion mimic phenotype, previously reported, was observed on leaves of NH1 overexpression plants [25]. To better compare the lesion mimic phenotypes between NH1 overexpression and OsWAK25 overexpression, we grew ten plants each of Kitaake, NH1 Ox 9A-5 (containing a transgenic copy of NH1 under the control of its native promoter in a Kitaake background), OsWAK25 Ox 2–6 and 3–11 under standard greenhouse conditions. All transgenic plants led to the development of these lesion mimic spots, similar in both timing and appearance. The lesions developed after approximately 4–5 weeks, once the plants reached the mature vegetative stage (before heading), and can be seen throughout the expanded leaves (Fig 5A).

After observing the lesion mimic phenotype, we hypothesized that OsWAK25 overexpression increases expression of defense-related genes. Leaf tissue was collected from 4 week-old plants grown under greenhouse conditions, and total RNA was extracted for qPCR analysis. *NH1* transcript is significantly increased in OsWAK25 overexpression lines when compared to wild type. Furthermore, defense related genes probenazole-inducible gene (*PBZ1*), phenylalanine ammonia lyase (*OsPAL2*), and pathogenesis-related gene 10 (*PR10*) are also upregulated (Fig 5B).

### Overexpression of OsWAK25 confers enhanced susceptiblity to necrotrophic pathogens, *Cochliobolus miyabeanus* and *Rhizoctonia solani*

**Fig 6:**
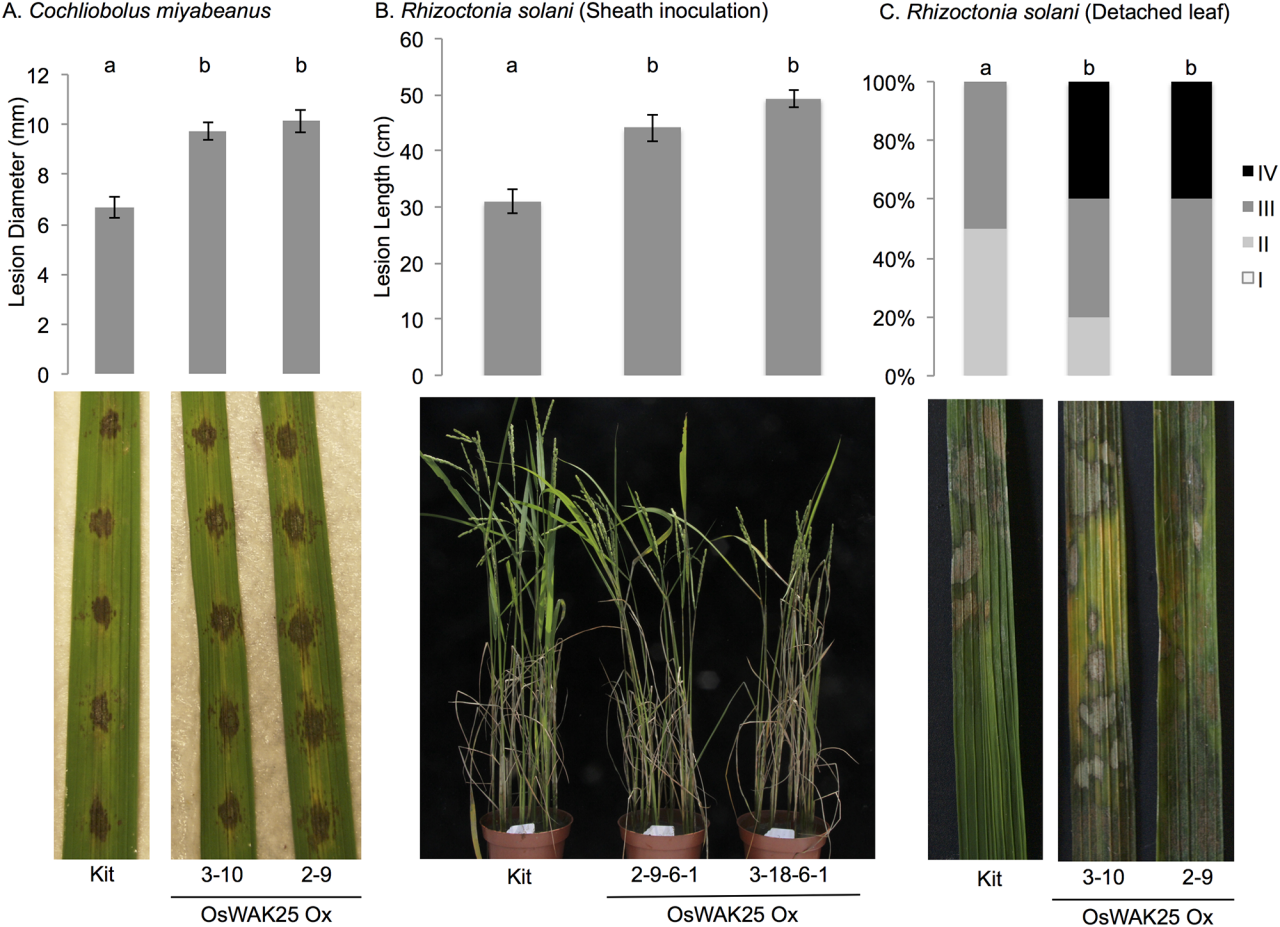
OsWAK25 overexpression plants are more susceptible to *Cochliobolus miyabeanus* and *Rhizoctonia solani*. (A) The fifth and sixth leaves were detached from six-week old plants and used for infection. Pictures and measurements were taken 3 dpi. (B) Photos and measurements were taken 14 dpi. *R*. *solani* sheath inoculation was conducted on six-week old plants. (C) Detached leaves from six-week old plants were infected with *R*. *solani* and disease leaf area (DLA) was scored after 3 days into 4 classes: Class I DLA < 25%, Class II 25% < DLA < 50%, Class III 50% < DLA <75%, Class IV DLA > 75%. Bars with different letters indicate statistical significance (p < 0.01) as determined by ANOVA with Tukey-Kramer post-hoc test for (A) and (B) and the Kruskal-Wallis test for (C). Both detached leaf experiments were conducted twice with similar results and data from one representative experiment shown. The sheath inoculation assay was conducted once.

To assess the effect of OsWAK25 overexpression on necrotrophic pathogen infection, we challenged the transgenic lines with fungal pathogens *Cochliobolus miyabeanus* and *Rhizoctonia solani*, the causal agents of brown spot and sheath blight diseases respectively. For both pathogens, the OsWAK25 overexpression lines exhibit increased disease symptoms compared to the control (Fig 6). For *Cochliobolus miyabeanus*, the diameter of disease lesions on lines 3–10 and 2–9 was significantly different from the Kitaake control, approximately 10 mm for OsWAK25 overexpression lines and 7 mm for Kitaake (Fig 6A). Both overexpression lines were also more susceptible to *Rhizoctonia solani*. Using a sheath inoculation method [26], lines 2-9-6-1 and 3-18-6-1 exhibited significantly longer lesions compared to Kitaake, approximately 45 cm for the overexpression lines and 31 cm for the control (Fig 6B). Finally, using a detached leaf assay, the area of infection was scored from I to IV, with I representing less than 25% infected area and IV representing greater than 75% infection (Fig 6C). Kitaake plants were scored as either II or III, whereas overexpression lines were scored as III or IV having more severe disease symptoms.

### Overexpression of XB15 compromises resistance to *Xoo* conferred by OsWAK25 overexpression

**Fig 7:**
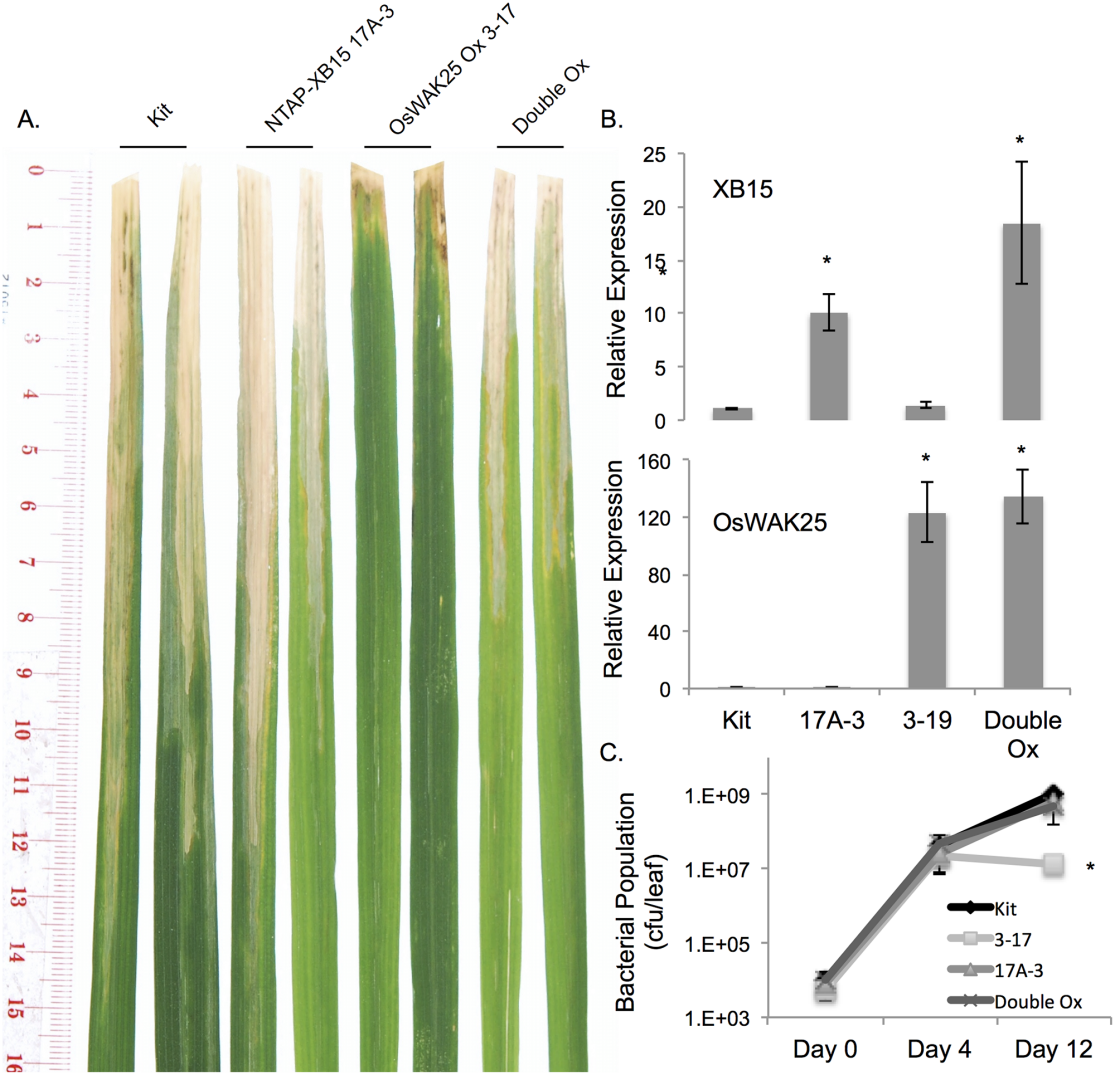
Overexpression of XB15 compromises OsWAK25-mediated resistance to *Xoo*. Transgenic lines NTAP-XB15 Ox 17A-3 and OsWAK25 Ox 3–17, both in the Kitaake background, were crossed and segregating F_2_ progeny of the double overexpression line were inoculated with *Xoo* strain PXO99 along with Kitaake and parental lines as controls. (A) Photos were taken 14 dpi. (B) Leaf tissue for RNA extraction was collected from 4-week old plants. Y-axes on the graphs indicate fold change from Kitaake. C_T_ values were first normalized to ubiquitin as an internal control followed by normalization to Kitaake. Each bar represents the average and standard error of 3 sibling plants. Asterisks represent statistical significance using Student’s t-test, p < 0.001. (C) Each time point represents the average and standard error of three sibling plants of three technical replicates each. The asterisk indicates statistical significance with Student’s t-test, p < 0.001. Each experiment was conducted three times with similarly significant results. See Fig 8 for segregation and lesion length data for F_2_ progeny.

**Fig 8:**
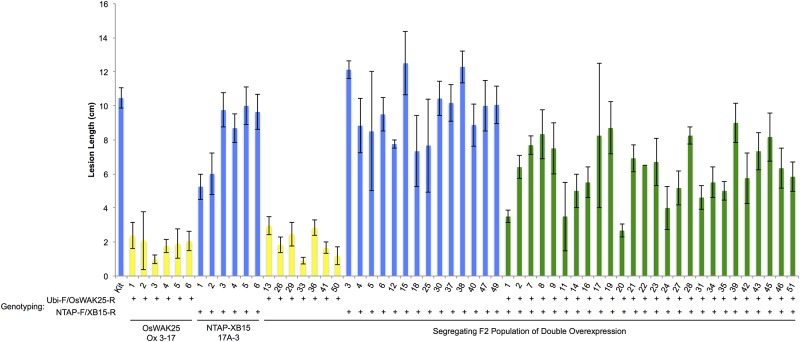
Genetic segregation of OsWAK25/XB15 double overexpression F_2_ progeny. 51 F_2_ progeny independently segregating for OsWAK25 overexpression and XB15 overexpression were challenged with PXO99 using scissors inoculation along with Kitaake and parental lines as controls. Lesions were measured 14 dpi. Each bar represents the average and standard error of at least three leaf measurements. Measurements were pooled by genotype and analyzed with an ANOVA followed by the Tukey-Kramer significance test. Different colors indicate statistical significance from the other genotypes.

In a protein-protein interaction study based on yeast two-hybrid experiments [19], the OsWAK25 kinase domain was shown to interact with XB15, a protein phosphatase 2C [27]. To test if overexpression of XB15 alters the resistance to *Xoo* conferred by overexpression of OsWAK25, we generated a double overexpression line by crossing OsWAK25 overexpression (OsWAK25 Ox 3–17) with a previously generated XB15 overexpression line, containing an N-terminal, TAP-tagged XB15 cDNA construct under a maize ubiquitin promoter also in the Kitaake background (NTAP-XB15 Ox 17A-3) [27]. We obtained four F_1_ hybrid plants from this cross, however only one contained both constructs by PCR analysis. This double overexpression hybrid was also confirmed to express both genes higher than the wild type controls (Fig 7B). We measured the lesion lengths and bacterial growth in the segregating F_2_ generation and compared these to the wild type control and the parental lines (Figs 7A and 8). The XB15 overexpression parental line and Kitaake control were susceptible to *Xoo*, displaying no significant differences in lesion lengths (approximately 8–10 cm). The OsWAK25 overexpression parental line had short lesions, about 2–3 cm. The lesion lengths for the double overexpression line exhibited an intermediate phenotype, statistically significant from either parental line (Fig 8). For the bacterial growth curve analysis shown in Fig 7C, the double overexpression lines were statistically similar to the Kitaake and XB15 overexpression lines. However, when the bacterial growth curve analysis was repeated, the double overexpression lines were intermediate between the Kitaake control and OsWAK25 overexpression, statistically significant from either group. Therefore, we conclude that the overexpression of XB15 compromises resistance to *Xoo* conferred by the overexpression of OsWAK25.

## Discussion

Wall-associated kinases are a family of proteins involved in a variety of growth and stress responses. We report that OsWAK25 contains a guanylyl cyclase motif [20] within the predicted intracellular kinase domain (Fig 1). Previous research has identified several *Arabidopsis* proteins that contain a similar dual domain [28], including AtWAKL10 that was shown to co-express with genes involved in the response to pathogens [9]. GC domains catalyze the formation of cGMP, a signaling molecule important for diverse physiological processes and stress responses, however the significance of the GC domain and the role of cGMP in plants are under debate [29–31].

A previous, microarray-based study published that *OsWAK25* expression is upregulated upon *M*. *oryzae* infection [17]. Our results provide additional information on the role of OsWAK25 in pathogen response. In this study, we further demonstrate that *OsWAK25* transcripts accumulate in response to wounding and BTH treatment (Fig 2). Overexpression of OsWAK25 results in the upregulation of defense response genes and confers resistance to *Xoo* and *M*. *oryzae*, both having hemibiotrophic lifestyles. Conversely, overexpression of OsWAK25 leads to increased susceptibility to *Cochliobolus miyabeanus* and *Rhizoctonia solani*, both necrotrophic fungal pathogens.

Similarly, NH1 is known to confer resistance to *Xoo* and *M*. *oryzae* [20, 25]. Following these data in addition to the upregulation of *NH1* in OsWAK25 overexpression plants and the accumulation of *OsWAK25* transcript following BTH treatment, we speculate that OsWAK25 is a positive regulator of NH1-dependent salicylic acid-mediated resistance. The enhanced resistance to hemibiotrophic pathogens we observe in OsWAK25 overexpression plants may be the result of activation of NH1-dependent SA-mediated pathways. In addition, the enhanced susceptibility we observe against necrotrophic fungal infection may be the result of SA-mediated suppression of other hormone pathways required for defense against such pathogens.

As a WAK-RLK, OsWAK25 is hypothesized to participate in signal transduction. In this classic scheme, kinases are activated by phosphorylation inducing a signal cascade that results in gene expression changes. A constitutively active kinase or overexpressed kinase can cause cell damage resulting in mimic disease symptoms, as seen in OsWAK25 overexpression. Tight regulation of these pathways is necessary to prevent cell damage; therefore the signal from a protein kinase is typically attenuated by an associated protein phosphatase. WAKs are no exception. In *Arabidopsis*, the PP2C, kinase-activated protein phosphatase (KAPP) has been shown to form a complex *in vivo* with WAK1 [32], and based on yeast-two hybrid protein interaction data, OsWAK25 interacts with XB15, a PP2C in rice [19]. In this study, we show that overexpression of XB15 compromises the resistance to *Xoo* conferred by OsWAK25 overexpression. Our phenotypic data supports the hypothetical *in vivo* interaction of these two proteins. In summary, our study presents a characterization of OsWAK25 in biotic stress responses and augments our knowledge about WAKs.

## Materials and Methods

### Amino acid sequence analysis

Protein domains were identified using the Simple Modular Architecture Research Tool (SMART) database (http://smart.embl-heidelberg.de/) and NCBI-CDD (http://www.ncbi.nlm.nih.gov/Structure/cdd/wrpsb.cgi). The GC domain was identified manually based on domain sequences given by Meier et al., 2010 [9].

### Rice transformation

Rice transformation was performed as described previously [22]. In brief, agrobacterium EHA105 was used to infect rice callus, and transformants of rice cultivar Kitaake carrying the OsWAK25 overexpression construct or RNAi construct were selected using hygromycin (S3 Fig).

### Plant growth conditions

For most experiments, rice seeds were surface sterilized with 70% ethanol and germinated on wet filter paper in light at 28°C. One week old seedlings were planted on Vegemix soil in a greenhouse and grown at 27–32°C with 40–60% humidity under natural sunlight supplemented by lamps set to a 16/8 hour on/off period when light levels fall below 600 W per square meter. Plants for *Xoo* inoculation were transferred from the greenhouse to a growth chamber set to a 14/10 hour light/dark photoperiod, a 28/24°C temperature cycle, and 85% constant humidity.

For fungal pathogen inoculations (not including *R*. *solani* sheath inoculation), plants were grown in a hydroponic gnotobiotic system. In brief, rice seeds were surface sterilized by agitation in 2% sodium hypochlorite for 20 minutes, rinsed three times with sterile demineralized water and germinated for 5 days at 28°C on wet filter paper. Transgenic WAK lines were selected in the presence of 50 μg/mL Hygromycin B. Germinated seeds were sown in sterilized vermiculite supplemented with half-strength Hoagland solution [33] and incubated for two weeks under growth chamber conditions (28°C day/24°C night, relative humidity: 60%, 12/12 light regimen). Thereafter, plants (3-leaf stage) were transferred to plastic containers containing modified Hoagland solution and grown for three weeks in the growth chamber. For *R*. *solani* sheath inoculation assay, plants were grown outside under natural conditions and were transferred to the greenhouse two days before inoculation.

### BTH treatment and wounding

Five-week old, greenhouse grown plants were sprayed with a 10 mM solution of Actigard (Syngenta) with 0.05% Tween 20 until leaves were coated evenly with droplets of solution. Wounding treatment was given to fully expanded leaves on five-week old plants by repeated puncturing with a needle along a 3–4 cm segment. For both assays, leaves were harvested into liquid nitrogen at the times indicated.

### RNA extraction and qPCR

Leaf tissue collected for analysis was frozen in liquid nitrogen and stored at -70°C until use. Total RNA was extracted using TRIzol reagent (Invitrogen) following the manufacturer’s instructions. Total RNA was treated with DNase I and purified with NucleoSpin RNA II kit (Macherey-Nagel). RNA quantity was measured on a Nanodrop (ND-1000 spectrophotometer) from Thermo Scientific. We used Superscript VILO cDNA synthesis kit (Invitrogen) for cDNA synthesis following manufacturer’s instructions. After quantification and dilution, 100 ng of cDNA was used as the template for each RT-PCR reaction using SsoFast EvaGreen Supermix (Bio-Rad) on a Bio-Rad CF96 Real-Time system coupled to a C1000 Thermal Cycler (Bio-Rad). Target gene expression was normalized to actin.

For fungal DNA quantification, amplifications were conducted on an optical 96-well plate using the Mx3005P real-time PCR detection system (Stratagene) and SYBR Green master mix (Stratagene/Bio-Connect). *M*. *oryzae* 28S rDNA was assayed in triplicate using 25 ng as input DNA and a passive reference dye (ROX) according to the manufacturer’s instructions (Stratagene). Two pools of DNA for each line were tested for DNA quantification. Each pool consisted of the youngest fully developed leaf from six individual plants.

### *Xoo* inoculation

Leaves from 5 to 6-wk-old rice plants were used for *Xoo* inoculation. *Xoo* strain, PXO99, was used to inoculate rice by the scissors-dip method [25]. To measure colony-forming units (CFU) from inoculated leaves, an entire leaf blade was cut into thin strips and suspended in 10 ml water to harvest bacteria. The suspension was diluted and plated on peptone sucrose agar (PSA) plates containing 20 mg/l cephalexin.

### Fungal pathogen inoculation

For *M*. *oryzae*, isolates VT5M1 and VT7 [23] were grown at 28°C on half-strength oatmeal agar (Difco). Seven-day-old mycelium was flattened onto the medium using a sterile spoon and exposed to blue light (combination of Philips TLD 18W/08 and Philips TLD 18W/33) for 7 d to induce sporulation. Upon sporulation, conidia were harvested as described by De Vleesschauwer et al. (2006) [34] and resuspended in 0.5% gelatin (type B from bovine skin; Sigma-Aldrich G-6650) to a final density of 1 × 10^4^ conidia per mL. For inoculation, 4-week-old seedlings (5-leaf stage) were misted with conidial suspension (1 mL per plant) using an artist airbrush powered by an air compressor. Immediately following inoculation, plants were placed in a dew chamber (28°C, 92% or greater relative humidity) for 18 hours to facilitate fungal penetration then transferred to a growth chamber (28°C, 12-h-light/12-h-dark). Six days after inoculation, the number of susceptible-type lesions, defined as elliptical to round lesions characterized by a gray center indicative of fungal sporulation, were counted [35, 36].

For *C*. *miyabeanus*, fifth and sixth stage leaves of 6-week-old plants were cut into 7-cm segments, placed in 14.5 × 14.5 cm Petri dishes lined with moist filter paper and drop inoculated with five, 15 μL droplets of conidial suspension (5 × 104 conidia mL^−1^ in 0.25% gelatin). Control leaves were mock inoculated with a 0.25% (w/v) gelatin suspension. After 24 h, the droplets were removed with a laboratory tissue, and at 72 hpi, lesions were measured.

For *R*. *solani* sheath inoculation assay, thin woody matchsticks (2–3 mm wide, 1 cm long and 1 mm thick) colonized by RH-9, a strain with strong virulence, were placed inside the third leaf sheath from the top of six-week old plants [26]. After inoculation, plants were placed in a tub covered with plastic with temperatures from 26–29°C and 70%—90% relative humidity. Lesions on the sheath were measured with a ruler 14 dpi. For the detached leaf assay, detached leaves were inoculated with a 0.8-cm-diameter mycelial disc of a 7-day-old PDA culture of *R*. *solani* strain MAN86. As a control, leaf segments were inoculated with a PDA plug without hyphae. Three days post inoculation, disease ratings were visually graded into four classes based on the leaf area affected; 1 = 0 to 25%, 2 = 26 to 50%, 3 = 51 to 75%, and 4 = more than 75% of leaf area affected.

### Crossing TAP-tagged XB15 overexpression and OsWAK25 overexpression lines

The transgenic line, OsWAK25 Ox 3–17, was used as the pollen recipient in a cross with pollen donor UBI::TAP:XB15 17A-3. Several seeds were obtained from this cross, yet one F_1_ plant was obtained containing both over-expression constructs, confirmed by PCR genotype and qPCR expression.

## Supporting Information

S1 Fig Summary of previously published protein-protein interactions with the OsWAK25 intracellular domain. OsWAK25 intracellular domain (red) interacts with several proteins. Black lines indicate yeast two-hybrid (Y2H) interaction; yellow lines indicate a Y2H interaction further validated by bimolecular fluorescence complementation (solid yellow if confirmed, or if not confirmed, dashed). Background color indicates the study, grey (Ding et al., 2009) and green (Seo et al., 2011).
(DOCX)

S2 Fig OsWAK25 annotated amino acid sequence. (DOCX)

S3 Fig OsWAK25 nucleotide sequence used for transgenic constructs. (DOCX)

S4 Fig OsWAK25 RNAi transgenic lines. Independently generated transgenic plants 2, 5, and 6 were confirmed by PCR (not shown) to contain the OsWAK25 RNAi pANDA construct. (A) These lines were tested for gene silencing with qPCR. Results indicate no statistically significant difference in expression from wild type, Kitaake based on Student’s t-test, p > 0.05. (B) Progeny from two lines, 2-6-9 and 5, were inoculated with three different strains of *Xoo* (PXO99, NXO256, and MXO90), with varying virulence on Kitaake rice. Lesion lengths were measured 14 dpi. Columns represent the average and standard error of pooled measurements from at least 3 separate plants. Line 2-6-9 exhibited statistically significant shorter lesions, Student’s t-test p = 0.0003, however this significance was not supported by bacterial population measurements. (C) Progeny from two lines, 2-6-9-5 and 6-2-NS-9, along with Kitaake controls were inoculated with *Xoo* strain, PXO99. Each time point represents the average and standard error of three sibling replicates, each having three technical replicates. No statistical significance was found.
(TIFF)

S1 Table List of primers used in this study. (XLSX)

## Abbreviations

BTH: benzothiadiazole
GC: guanylyl cyclase
RLK: receptor-like kinase
SA: salicylic acid
SAR: systemic acquired resistance
WAK: wall-associated kinase
*Xoo*: *Xanthomonas oryzae* pv. *oryzae*
