# Supplementary material for: Overexpression of Rice Wall-Associated Kinase 25 (OsWAK25) Alters Resistance to Bacterial and Fungal Pathogens
Source: PLoS One. 2016 Jan 21;11(1):e0147310. doi: 10.1371/journal.pone.0147310 (PMC4721673; doi:10.1371/journal.pone.0147310)
Supplement: S1 Fig — OsWAK25 intracellular domain (red) interacts with several proteins. Black lines indicate yeast two-hybrid (Y2H) interaction; yellow lines indicate a Y2H interaction further validated by bimolecular fluorescence complementation (solid yellow if confirmed, or if not confirmed, dashed). Background color indicates the study, grey (Ding et al., 2009) and green (Seo et al., 2011). (DOCX) [file pone.0147310.s001.docx]

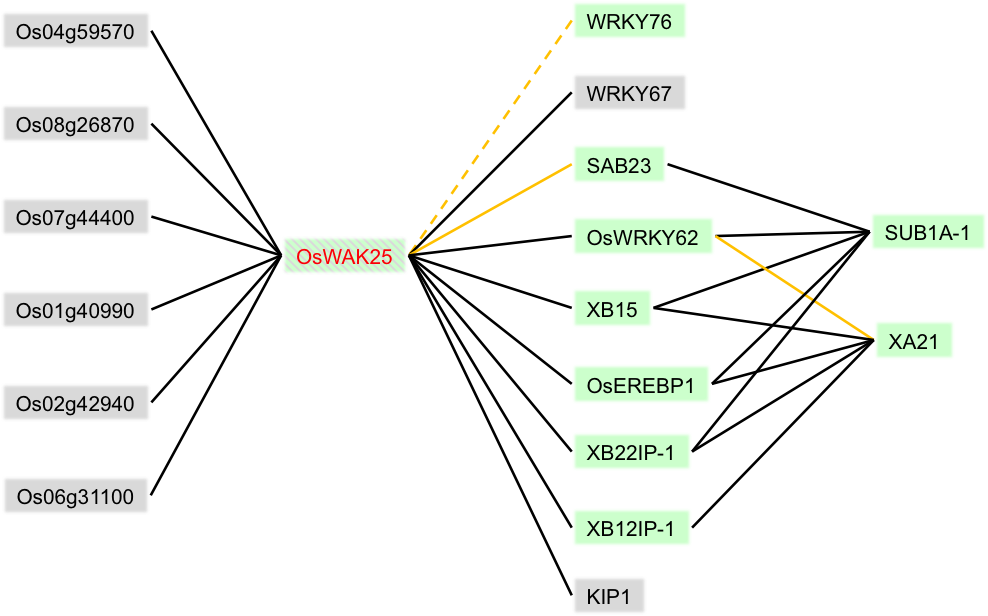


Compilation of protein-protein interactions with OsWAK25 intracellular domain (red). Black lines indicate yeast two-hybrid (Y2H) interaction; yellow lines indicate a Y2H interaction that was then tested with bimolecular fluorescence complementation (solid yellow if confirmed, or if not confirmed, dashed).

Background color indicates the study, grey (Ding et al., 2009) and green (Seo et al., 2011).

| **Locus ID** | **Gene Name** | **MSU rice genome annotation** | **Source** |
| --- | --- | --- | --- |
| LOC_Os03g12470 | OsWAK25 | OsWAK receptor-like protein kinase, expressed |  |
| LOC_Os10g28610 | KIP1 | KIP1, putative, expressed | Ding et al., 2009 |
| LOC_Os09g25060 | OsWRKY76 | WRKY76, expressed | Ding et al., 2009, Seo et al., 2011 |
| LOC_Os12g32980 | SAB23 | potyvirus VPg interacting protein, putative, expressed | Ding et al., 2009, Seo et al., 2011 |
| LOC_Os04g59570 |  | expressed protein | Ding et al., 2009 |
| LOC_Os08g26870 |  | wound responsive protein, putative, expressed | Ding et al., 2009 |
| LOC_Os07g44400 |  | POK1, putative, expressed | Ding et al., 2009 |
| LOC_Os01g40990 |  | expressed protein | Ding et al., 2009 |
| LOC_Os02g42940 |  | MSP domain containing protein, expressed | Ding et al., 2009 |
| LOC_Os06g31100 |  | E1-BTB2 - Bric-a-Brac, Tramtrack, and Broad Complex domain with E1 subfamily conserved sequence, expressed | Ding et al., 2009 |
| LOC_Os09g25070 | OsWRKY62 | WRKY62, expressed | Seo et al., 2011 |
| LOC_Os05g49700 | XB12IP-1 | AP2 domain containing protein, expressed | Seo et al., 2011 |
| LOC_Os05g09020 | OsWRKY67 | WRKY67, expressed | Seo et al., 2011 |
| LOC_Os11g01550 | XB22IP-1 | DUF260 domain containing protein, putative, expressed | Seo et al., 2011 |
| LOC_Os02g54160 | OsEREBP1 | AP2 domain containing protein, expressed | Seo et al., 2011 |
| LOC_Os03g60650 | XB15 | protein phosphatase 2C, putative, expressed | Seo et al., 2011 |
