# Supplementary material for: Overexpression of Rice Wall-Associated Kinase 25 (OsWAK25) Alters Resistance to Bacterial and Fungal Pathogens
Source: PLoS One. 2016 Jan 21;11(1):e0147310. doi: 10.1371/journal.pone.0147310 (PMC4721673; doi:10.1371/journal.pone.0147310)
Supplement: S2 Fig — (DOCX) [file pone.0147310.s002.docx]

**Supplementary Figure 2.** OsWAK25 annotated amino acid sequence

Protein Sequence, [LOC_Os03g12470.1](http://rice.plantbiology.msu.edu/cgi-bin/ORF_infopage.cgi?orf=13103.m01428):

MRGAARLLLPLVVLLLHAARGSAGSTGGGGNGSCTQSCGRMRVPYPFGFSRGCTVQLGCD DASGTAWLGGTRGLGLLVSNVTPRAIVLTLPPNCSRPLNESLDALFTDNYAPTAQNALVV SSCDPQAAARLSNCSIPPEAYLEKSCNSIRCVLPSTKANVDGTNVTDPFLNRSEMRRLGS DCRGLVSASIYSNTAGPALQLTALELDWWVQGRCGCSSHAICDGFTPPSTQKEAFRCECQ EGFEGDGYTAGAGCRRVPKCNPSKYLSGSCGKLVQIGLLVAGVFFGAMVMGITCLVYHLL RRRSAALRSQKSTKRLLSEASCTVPFYTYREIDRATNGFAEDQRLGTGAYGTVYAGRLSN NRLVAVKRIKQRDNAGLDRVMNEVKLVSSVSHRNLVRLLGCCIEHGQQILVYEFMPNGTL AQHLQRERGPAVPWTVRLRIAVETAKAIAYLHSEVHPPIYHRDIKSSNILLDHEYNSKVA DFGLSRMGMTSVDSSHISTAPQGTPGYVDPQYHQNFHLSDKSDVYSFGVVLVEIITAMKA VDFSRVGSEVNLAQLAVDRIGKGSLDDIVDPYLDPHRDAWTLTSIHKVAELAFRCLAFHS EMRPSMAEVADELEQIQVSGWAPSTDDATFMSTTSSLCSSAPSRCTDKSWGTAKSKRQAA ANAVVKQETTKCAVADSPVSVQERWFSDRSSPSSNSLLRNSSLN*

Yellow-EGF_CA domain

Pink: Transmembrane domain

Red: Kinase domain

Grey: Catalytic domain

Blue: GC domain (Guanylyl Cyclase)

Green: Signal Peptide

Protein length: 705 aa

Nucleotide: 2115 bp

Mol Wt: 76160 dalton

PI: 8.26
