# Supplementary material for: Overexpression of Rice Wall-Associated Kinase 25 (OsWAK25) Alters Resistance to Bacterial and Fungal Pathogens
Source: PLoS One. 2016 Jan 21;11(1):e0147310. doi: 10.1371/journal.pone.0147310 (PMC4721673; doi:10.1371/journal.pone.0147310)
Supplement: S3 Fig — (DOCX) [file pone.0147310.s003.docx]

**Supplementary Figure 3.** OsWAK25 nucleotide sequence used for transgenic constructs

Full length CDS was used for the construction of overexpression lines using Ubi-NC1300RFCA. The sequence highlighted in yellow was used for the pANDA RNAi construct (Seo et al., 2011).

ATGCGGGGCGCGGCGCGGCTACTCCTGCCACTGGTGGTGCTGCTGCTGCACGCAGCACGC GGATCAGCGGGATCGACGGGCGGCGGAGGCAACGGCAGCTGCACGCAGAGCTGCGGCCGC ATGAGGGTGCCGTACCCGTTCGGCTTCTCCAGAGGCTGCACGGTTCAGCTCGGCTGCGAC GACGCCTCCGGCACCGCGTGGCTCGGCGGGACGCGCGGGCTGGGCCTGCTCGTGAGCAAC GTGACGCCGCGCGCCATCGTCCTCACCCTGCCCCCCAACTGCTCCCGCCCGCTCAACGAG TCCCTGGATGCGCTCTTCACCGACAACTACGCGCCCACCGCGCAGAACGCCCTGGTCGTG AGCTCGTGCGACCCGCAGGCCGCCGCCCGCCTCAGCAACTGCAGCATCCCACCCGAGGCC TACCTCGAGAAGAGCTGCAATTCCATCCGCTGCGTCTTACCTTCTACCAAAGCCAACGTC GACGGGACAAACGTCACAGACCCTTTCTTGAATAGAAGCGAGATGCGGCGGCTCGGCTCG GACTGCCGCGGGCTCGTGTCGGCGTCGATCTATTCGAACACGGCGGGGCCGGCGCTGCAG CTGACCGCGCTGGAGCTGGATTGGTGGGTGCAGGGGCGGTGCGGCTGCTCGAGCCACGCC ATCTGCGACGGGTTCACCCCGCCGTCTACGCAGAAGGAGGCGTTCCGGTGCGAGTGCCAG GAGGGGTTCGAGGGCGACGGCTACACCGCCGGCGCCGGTTGCCGGAGAGTTCCAAAGTGT AATCCTTCAAAATACCTATCAGGATCATGTGGCAAGTTGGTTCAGATCGGCCTTCTTGTG GCAGGAGTCTTTTTTGGAGCCATGGTGATGGGCATCACCTGCTTGGTGTACCACCTGCTG CGGCGCCGGTCGGCGGCCCTCCGGAGCCAGAAGAGCACGAAGCGGCTGCTGTCGGAGGCG TCCTGCACGGTGCCCTTCTACACGTACCGCGAGATCGATCGCGCCACCAACGGCTTCGCC GAGGACCAGCGCCTTGGCACGGGCGCGTACGGCACGGTGTACGCGGGGCGGCTGAGCAAC AACCGCCTCGTGGCCGTGAAGCGGATCAAGCAGCGCGACAACGCCGGGCTGGACCGCGTG ATGAACGAGGTGAAGCTCGTGTCGTCGGTGAGCCACCGCAACCTCGTCCGCCTCCTCGGC TGCTGCATCGAGCACGGGCAGCAGATCCTCGTCTACGAGTTCATGCCCAACGGCACGCTG GCGCAGCACCTGCAGCGGGAGCGCGGCCCGGCCGTGCCGTGGACGGTCCGCCTCCGCATC GCCGTCGAGACGGCCAAGGCCATCGCGTACCTGCACTCGGAGGTGCACCCGCCCATCTAC CACCGCGACATCAAGTCCAGCAACATCCTGCTCGACCACGAGTACAACTCCAAGGTCGCC GACTTCGGGCTGTCGCGGATGGGCATGACGTCCGTCGACTCGTCGCACATCTCCACCGCG CCGCAGGGCACGCCGGGGTACGTCGACCCTCAGTACCACCAGAACTTCCACCTCTCGGAC AAGAGCGACGTGTACAGCTTCGGCGTCGTGCTCGTCGAGATCATCACGGCCATGAAGGCC GTCGACTTCAGCCGGGTTGGCAGCGAGGTCAACCTGGCGCAGCTGGCCGTCGACAGGATC GGGAAAGGCAGCCTCGACGACATCGTCGACCCCTACCTAGACCCGCACAGGGACGCCTGG ACTCTCACGTCCATCCACAAGGTGGCCGAGCTGGCGTTTCGGTGCCTGGCGTTCCACAGC GAGATGAGACCTTCCATGGCTGAGGTCGCCGACGAGCTGGAACAGATTCAGGTCAGCGGG TGGGCGCCGTCCACGGATGACGCCACATTCATGTCAACGACGTCCTCGCTTTGCTCGTCG GCTCCATCACGTTGCACGGACAAGTCTTGGGGGACCGCTAAGAGCAAGAGGCAGGCCGCG GCAAACGCAGTGGTAAAGCAAGAGACGACGAAGTGTGCAGTCGCCGACTCCCCCGTGTCC GTGCAGGAGAGATGGTTCAGCGATAGGAGCTCCCCTTCCTCAAATAGCCTGCTGAGGAAT

AGCTCCCTGAACTAA

**
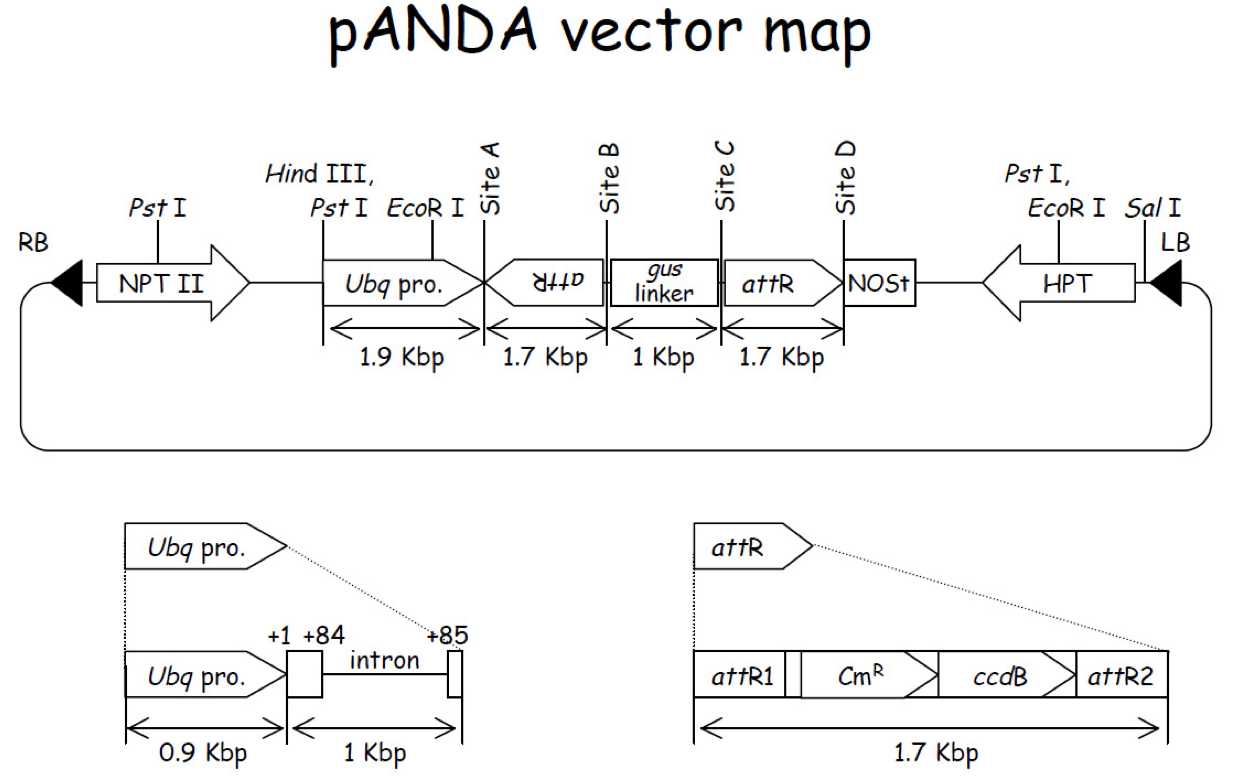

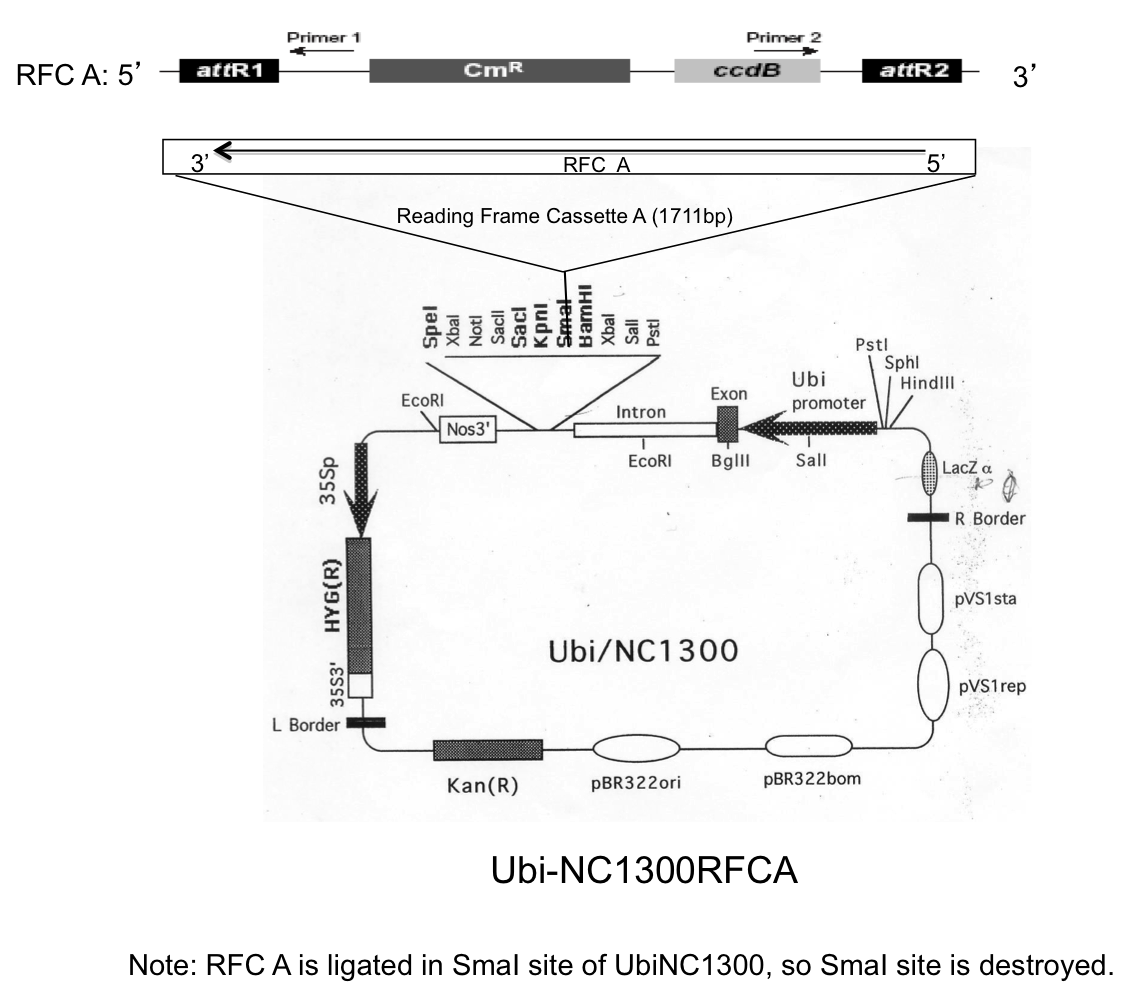
**
