# Supplementary figures and images for: Overexpression of Rice Wall-Associated Kinase 25 (OsWAK25) Alters Resistance to Bacterial and Fungal Pathogens
Source: PLoS One. 2016 Jan 21;11(1):e0147310. doi: 10.1371/journal.pone.0147310 (PMC4721673; doi:10.1371/journal.pone.0147310)

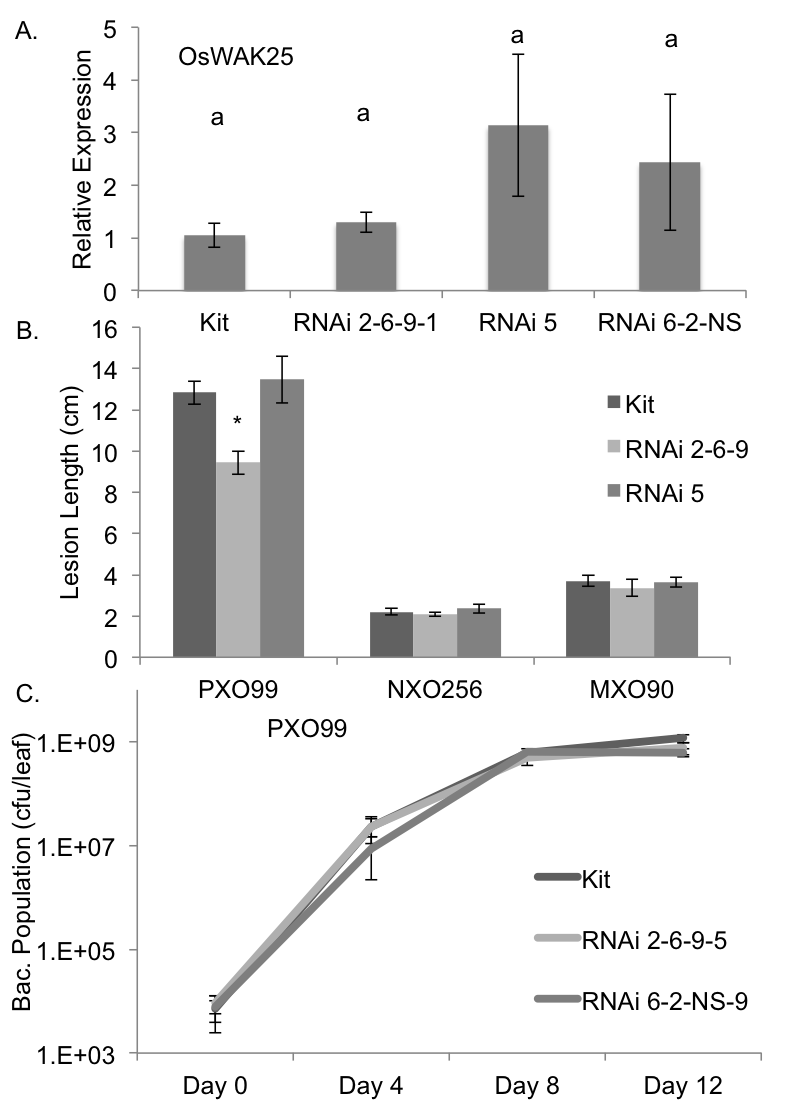

Supplement: S4 Fig — Independently generated transgenic plants 2, 5, and 6 were confirmed by PCR (not shown) to contain the OsWAK25 RNAi pANDA construct. (A) These lines were tested for gene silencing with qPCR. Results indicate no statistically significant difference in expression from wild type, Kitaake based on Student’s t-test, p > 0.05. (B) Progeny from two lines, 2-6-9 and 5, were inoculated with three different strains of Xoo (PXO99, NXO256, and MXO90), with varying virulence on Kitaake rice. Lesion lengths were measured 14 dpi. Columns represent the average and standard error of pooled measurements from at least 3 separate plants. Line 2-6-9 exhibited statistically significant shorter lesions, Student’s t-test p = 0.0003, however this significance was not supported by bacterial population measurements. (C) Progeny from two lines, 2-6-9-5 and 6-2-NS-9, along with Kitaake controls were inoculated with Xoo strain, PXO99. Each time point represents the average and standard error of three sibling replicates, each having three technical replicates. No statistical significance was found. (TIFF) [file pone.0147310.s004.tiff]
